# The Emerging Role of Ferumoxytol-Enhanced MRI in the Management of Cerebrovascular Lesions

**DOI:** 10.3390/molecules18089670

**Published:** 2013-08-13

**Authors:** Nohra Chalouhi, Pascal Jabbour, Vincent Magnotta, David Hasan

**Affiliations:** 1 Department of Neurosurgery, Thomas Jefferson University and Jefferson Hospital for Neuroscience, Philadelphia, PA 19107, USA; E-Mails: nohra.chalouhi@jefferson.edu (N.C.); pascal.jabbour@jefferson.edu (P.J.); 2 Department of Neurosurgery, University of Iowa, Iowa City, IA 52242, USA; E-Mail: vincent-magnotta@uiowa.edu

**Keywords:** aneurysm, ferumoxytol, arteriovenous malformation, magnetic resonance imaging, inflammation

## Abstract

Inflammation is increasingly being understood to be a key component to the pathophysiology of cerebrovascular lesions. Ferumoxytol, an iron oxide nanoparticle coated by a carbohydrate shell, has been used in MRI studies as an inflammatory marker because it is cleared by macrophages. Ferumoxytol-enhanced MRI has emerged as an important tool for noninvasive assessment of the inflammatory status of cerebrovascular lesions, namely aneurysms and arteriovenous malformations. Moreover, preliminary evidence suggests that ferumoxytol-enhanced MRI could be applied as a non-invasive tool to differentiate “unstable” lesions that require early intervention from “stable” lesions in which observation may be safe. Assessment of the effects of anti-inflammatory pharmacological interventions on cerebrovascular lesions is also a potentially crucial application of the technique. Future improvements in technique and MRI signal quantification will certainly pave the way for widespread and efficient use of ferumoxytol-enhanced MRI in clinical practice. In this paper, we review current data regarding ferumoxytol-enhanced MRI and discuss its current/potential applications and future perspectives.

## 1. Introduction

Intracranial aneurysms (IAs) and arteriovenous malformations (AVMs) account for the large majority of cerebrovascular lesions encountered in practice [1]. IAs are responsible for 85% of subarachnoid hemorrhages (SAHs) while AVMs account for 5%–9% of all cases [2,3]. Despite considerable advances in diagnostic methods, surgical techniques, and peri-operative management, the case fatality rate for patients with SAH is as high as 50% [3,4,5]. Moreover, 50% of those who survive remain disabled and do not return to their previous activities. Preventing IA/AVM rupture is therefore the main goal of treatment. 

Because current therapeutic strategies including microsurgical and endovascular treatment are not innocuous [6,7,8,9,10,11], it is important to balance the risk of morbidity and mortality that may result from treatment against the natural history of unruptured IAs and AVMs. Unfortunately, the natural history of these lesions remains uncertain making the decision to intervene or not a difficult and somewhat subjective one [1]. Factors commonly taken into account when contemplating treatment for IAs include patient age, patient preference, size/morphology of aneurysm, risk factors (smoking, arterial hypertension *etc.*), location of aneurysm, personal or family history of SAH, and physician judgment. Although important, these factors cannot be relied upon for guiding therapy in all patients. For example, while there is little doubt that a 30 mm aneurysm in a 35-year-old patient with a family history of SAH should be treated, it is uncertain whether a 75-year-old patient with a 5 mm aneurysm arising from the middle cerebral artery benefits at all from treatment. With regard to unruptured AVMs, it is debated whether an intervention improves patient outcomes [3,12,13,14]. Therefore, non-invasive tools to differentiate “unstable” cerebrovascular lesions that require early intervention from “stable” lesions in which observation is safe are needed. 

Ferumoxytol-enhanced MRI constitutes today the most promising and exciting tool for guiding the management of cerebrovascular lesions. The principle behind this method is based on the critical role of inflammation in general and macrophages in particular in the formation and rupture of IAs and AVMs [15,16,17]. Our group has been investigating the feasibility and utility of ferumoxytol-enhanced MRI in IAs and AVMs. In the present paper, we review current data regarding ferumoxytol-enhanced MRI in IAs and AVMs and discuss potential applications, technical aspects, and future perspectives.

## 2. Inflammation and Macrophages in IAs and AVMs

It has become clear that inflammation underlies the formation and ultimately the rupture of IAs [15,18,19,20]. The common pathway for IA formation involves endothelial dysfunction, a mounting inflammatory response with vascular smooth muscle cell phenotypic modulation, extracellular matrix remodeling, and subsequent cell death and vessel wall degeneration. Hemodynamic stress at arterial bifurcations, arterial junctions, or abrupt vascular angles elicits a series of proinflammatory changes in endothelial cells [21]. Indeed, Jamous *et al*. [22,23]. found that endothelial cell injury was the earliest change in aneurysm wall, followed by the formation of an inflammatory zone that leads to proteolytic destruction of the vascular extracellular matrix by metalloproteinases and ultimately to aneurysm formation. Endothelial injury and the disruption of endothelial tight junctions is also associated with the migration of leukocytes mainly macrophages into aneurysm walls [24]. In fact, in response to hemodynamic stress, endothelium undergoes several proinflammatory changes [25] including activation of nuclear factor kappa B (NF-kB) [26], monocyte chemoattractant protein 1 (MCP-1) [27,28] and vascular cell adhesion molecule 1 (VCAM-1) [29,30], all of which are highly chemotactic to macrophages. The central role of macrophages in the pathogenesis of IAs has been well documented [31,32]. Macrophage depletion and knockout of the MCP-1 gene halts IA formation in animal models [20]. Macrophages are thought to exert this deleterious action through the release of extra cellular matrix-degrading proteolytic enzymes, pro-inflammatory cytokines, and induction of apoptosis of smooth muscle cells [31,32]. Macrophages are a major source of metalloproteinases which digest arterial wall extracellular matrix and cause further damage via upregulation of other proteinases and angiogenic factors [33,34,35,36,37]. Moreover, the levels of metalloproteinases are higher in ruptured than in unruptured aneurysms suggesting that macrophage-induced breakdown of vessel extracellular matrix underlines IA rupture [38]. We have recently demonstrated that macrophages are expressed in the wall of human IAs and that the two major subsets of macrophages namely M1 and M2 cells, which play opposite roles during inflammation, are present in equal proportions in unruptured aneurysms [39]. In ruptured aneurysms, however, this critical M1/M2 balance is lost in favor of M1 cells (proinflammatory) suggesting that a polarized pro-inflammatory response involving M1 macrophages plays a key role in the cascade of events leading to aneurysm rupture. 

As for IAs, inflammation is also thought to contribute to the pathophysiology of AVMs. Higher levels of inflammatory cytokines including interleukin-6 and matrix metalloproteinases were found in AVMs compared with normal brain [40,41]. In AVM specimens, macrophages and other inflammatory cells can be seen in the vascular walls and intervening stroma [42]. Chen *et al.* [43] found increased expression of macrophage migration inhibitory factor, matrix metalloproteinase 9, and cleaved caspase-3 in AVM specimens of patients who did not received preoperative embolization. Along similar lines, Aziz *et al.* [44] detected activation of NF-κB in the endothelium and perivascular infiltrating inflammatory cells within the cerebral AVM nidus. Additionally, polymorphisms in interleukin 6, interleukin-1beta, and TNF-alpha were found to be associated with intracerebral hemorrhage in the natural course of AVMs [45,46,47]. 

Given the strong link between inflammation/macrophages and the risk of IA and AVM rupture, a noninvasive means to detect inflammation/macrophages is valuable for identifying IAs and AVMs at risk of hemorrhage.

## 3. Ferumoxytol-Enhanced MRI

Ferumoxytol (AMAG Pharmaceuticals, Lexington, MA, USA), an iron oxide nanoparticle coated by a carbohydrate shell, is a member of the class of nanoparticles known as ultrasmall superparamagnetic iron oxide (USPIOs). The drug was approved by the Food and Drug Administration in 2009 as a treatment for iron deficiency anemia in patients with chronic renal failure (dosing 510 mg IV once, then repeat 3–8 days later) [48,49]. Ferumoxytol is contraindicated in patients with iron overload. Monitoring for hypotension and hypersensitivity reactions is warranted for at least 30 min after injection [50]. Secondary effects may rarely occur and include hypotension, constipation, dizziness, peripheral edema, diarrhea, and nausea. Ferumoxytol is a pregnancy category C drug [50].

**Figure 1:**
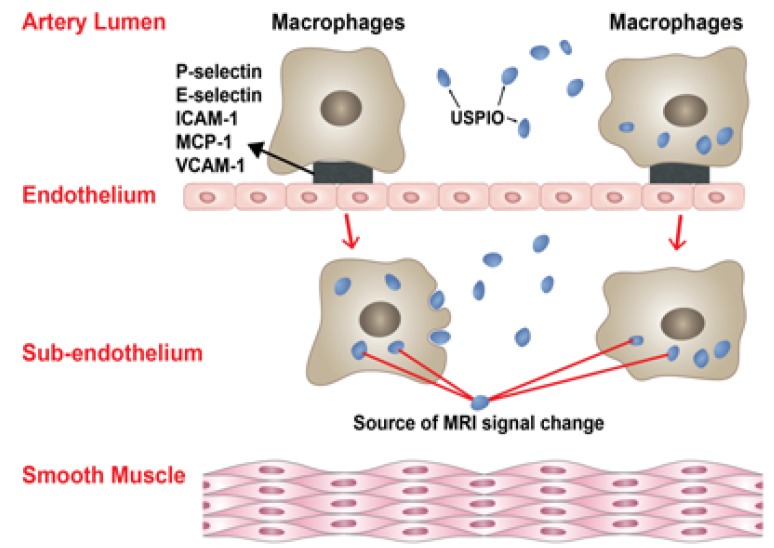
The insert illustrates the two mechanisms by which macrophages and USPIOs result in MRI signal changes in the walls of human cerebral aneurysms. Uptake of USPIOs by monocytes may occur in the arterial lumen or in the subendothelium.

In a magnetic field, ferumoxytol is magnetized generating field gradients. Ferumoxytol appears hypointense on MRI T2* weighted gradient-echo (GE) sequences and can appear hyperintense on T1 weighted spin-echo sequences. The drug can be visualized intravascularly for up to 72 h but begins to clear within 24 h; delayed visualization is secondary to macrophage-uptake and occurs within 24 h. Uptake of ferumoxytol by monocytes may occur in the arterial lumen or in the subendothelium (Figure 1).

Here it should be mentioned that ferumoxytol can only be used as an “off label” drug for MRI applications. Again, the drug is only approved for use in the US as feraheme, a parenteral drug for iron deficiency in patients with chronic renal failure. It should not be concluded from this paper that we recommend the routine off label use of ferumoxytol for MRI since severe and sometimes fatal adverse events may occur [51].

Ferumoxytol has been used in MRI studies for cardiovascular imaging, endoleak detection in patients with aortic stent grafts, depiction of deep vein thrombosis, tumor progression, and cancer staging among other applications [50,52,53,54,55,56,57,58,59,60]. It also is useful as an inflammatory marker, when imaging is delayed because it is cleared by macrophages [56,59]. Although ferumoxytol may differ from other USPIOs with regard to coatings, blood circulation times, and affinity to macrophages, several studies in experimental animals and humans have demonstrated that USPIO accumulates in atherosclerotic plaques in the abdominal aorta and internal carotid artery [61,62,63,64,65,66,67] Ruehm *et al.* [63] elegantly demonstrated that USPIOs are phagocytosed by macrophages in atherosclerotic plaques of the aortic wall of hyperlipidemic rabbits in a quantity sufficient to cause susceptibility effects detectable by MRI. Along similar lines, Hyafil *et al.* [62] showed that USPIOs massively accumulate in neointimal macrophages in the aortic wall of hypercholesterolemic rats. Moreover, they demonstrated that MRI signal generated by USPIO strongly correlated with macrophage infiltration in aortic tissue. Thus, USPIO-enhanced MRI allows noninvasive assessment of the inflammatory status of vascular lesions through the detection of activity of macrophages. 

## 4. Ferumoxytol-Enhanced MRI in IAs

Our initial work focused on demonstrating the feasibility and optimal parameters of imaging macrophages in the wall of human cerebral aneurysms using ferumoxytol-enhanced MRI [68]. For this purpose, 19 unruptured aneurysms in 11 patients were imaged with T2-GE–MRI sequence using two protocols. The first protocol consisted of an infusion of 2.5 mg/kg of ferumoxytol and imaging at day 0 and 1, while the second protocol consisted of an infusion of 5 mg/kg of ferumoxytol and imaging at day 0 and 3. Ferumoxytol-associated signal changes were noted in 50% of aneurysm walls with the first protocol *versus* 78% with the second protocol. Beyond 5 days, ferumoxytol is no longer detectable in tissue. Thus, the optimal technique for imaging macrophages in aneurysm walls is infusion of 5 mg/kg of ferumoxytol and imaging at 72 h after injection. Interestingly, the two aneurysms that did not demonstrate ferumoxytol-associated loss of signal intensity with this protocol were found to have significant calcification during surgical clipping. Importantly, aneurysm tissue harvested from patients infused with ferumoxytol stained positive for CD68 and Prussian blue while tissue harvested from controls stained positive for CD68 but not for Prussian blue. The findings suggested that iron nanoparticles were not inherently found in aneurysm walls but that there was active uptake of these particles by macrophages in aneurysm walls. This proof-of-principle study established the optimal protocol for ferumoxytol-enhanced MRI and demonstrated the possibility of imaging the inflammatory response in aneurysm walls using macrophages as a surrogate marker. Of note, the long delay between IV injection and optimal imaging time point might be a disadvantage in clinical work flow.

**Figure 2:**
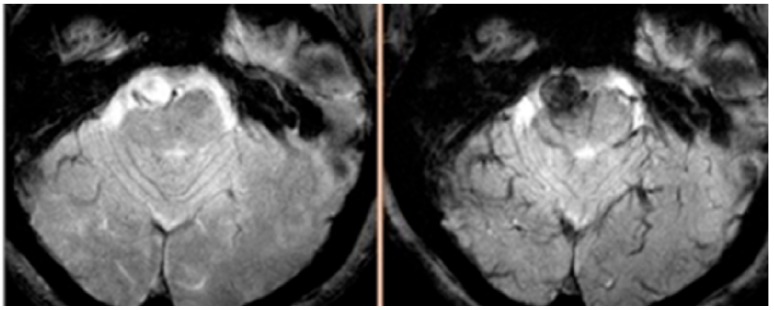
MRI, T2* GE sequence, from a patient showing early uptake of ferumoxytol. The aneurysm ruptured within three months.

In the December 2012 issue of the journal *Stroke*, a thought-provoking original article entitled “Early Change in Ferumoxytol-Enhanced Magnetic Resonance Imaging Signal Suggests Unstable Human Cerebral Aneurysm. A Pilot Study” was published [69]. In this article, Hasan *et al.* [69] demonstrated that the findings of ferumoxytol-enhanced MRI may predict the risk of aneurysm rupture. The findings were a major advance because the prospect of determining the risk of IA rupture using imaging techniques was previously unimaginable. Briefly, 30 unruptured aneurysms were imaged with MRI 24 h after infusion of ferumoxytol while 18 aneurysms were imaged 72 h post-ferumoxytol. All aneurysms with early uptake of ferumoxytol (at 24 h) that were managed conservatively ruptured within six months. In contrast, none of the aneurysms with late uptake of ferumoxytol (at 72 h) ruptured or increased in size after six months. Importantly, expression of cyclooxygenase-1, microsomal-prostaglandin-E2 synthase-1, and macrophages was similar in unruptured aneurysms with early uptake of ferumoxytol and ruptured aneurysms. Moreover, expression of these inflammatory molecules was significantly higher in aneurysms with early uptake of ferumoxytol *versus* aneurysms with late uptake. The findings of this study provided evidence that aneurysms with early uptake of ferumoxytol on MRI were prone to rupture and thus may warrant early operative intervention. In fact, early MRI signal changes noted on T2*GE sequence are produced by increased uptake of ferumoxytol nanoparticles by macrophages that are localized within aneurysm walls (Figure 2). Early uptake therefore suggests an active inflammatory process, as demonstrated histopathologically. This study opened new horizons for physicians treating intracranial aneurysms. As such, the technique could be applied in clinical practice as a non-invasive tool to differentiate “unstable” aneurysms that require early intervention from “stable” aneurysms in which observation may be safe. Specifically, ferumoxytol-enhanced MRI could be valuable in identifying rupture-prone aneurysms in patients who pose a therapeutic dilemma, namely elderly patients and patients harboring small aneurysms [1]. Additionally, ferumoxytol-enhanced MRI allowed us to provide the first direct evidence that inflammation was a causal factor in progression of cerebral aneurysms in humans to rupture as previous studies had fallen short of demonstrating that the inflammatory response was present before rupture of aneurysms, as opposed to a response to aneurysm rupture [13]. At the plenary session of the American Association of Neurological Surgeons (AANS) 81st Annual Scientific Meeting, where the findings of the study were presented, IA experts agreed on the importance of this work while noting that a larger study was needed. Commenting on the study, Adel Malek, MD, Director of the Cerebrovascular and Endovascular Division in the Department of Neurosurgery at Tufts University School of Medicine concluded that this imaging technique “may offer the needed noninvasive metric to help stratify rupture risk and guide optimal decision-making.” Lastly, it should be noted that because early uptake of ferumoxytol indicates IA instability, delayed imaging beyond 24 h may probably become unnecessary for assessing the risk of aneurysm rupture. Imaging at 72 h, however, may still be useful for other indications of the technique (such as assessment of effect of therapy) since it represents the optimal timing for imaging macrophages in IA walls.

Building on our previous work on ferumoxytol-enhanced MRI, we assessed in a small study whether the technique can be used to noninvasively measure the effects of anti-inflammatory pharmacological interventions on IAs [70]. Specifically, the goal of the study was to image aspirin effect on macrophages in the wall of human cerebral aneurysm using ferumoxytol-enhanced MRI, as daily intake of aspirin had been previously shown to reduce the incidence of cerebral aneurysm rupture [71]. Five patients with known intracranial aneurysms underwent baseline imaging using ferumoxytol-enhanced MRI before they received 325 mg aspirin p.o daily for three months. After three months, imaging studies were repeated and showed decreased signal intensity on ferumoxytol-enhanced MRI in aneurysm walls compared to the baseline images in all five patients. These results demonstrated that ferumoxytol-enhanced MRI allows imaging the effect of antinflammatory interventions on IAs. The findings also provided further evidence that aspirin decreases inflammation in the wall of IAs. 

Using ferumoxytol-enhanced MRI and immunostaining, we tested in a randomized controlled trial the effects of aspirin on inflammatory cells and molecules in the wall of human cerebral aneurysms [72]. Eleven patients harboring unruptured intracranial aneurysms were randomized into aspirin-treated (81 mg daily) (n = 6) and untreated (control) groups (n = 5). Aneurysms were imaged at baseline using ferumoxytol-enhanced MRI to estimate uptake by macrophages and re-imaged three months later before undergoing microsurgical clipping. The signal intensity in aspirin treated patients was decreased in the wall of cerebral aneurysms on ferumoxytol-enhanced MRI after three months of aspirin treatment whereas in the control group signal intensity did not change after three months of observation. Importantly, the findings of ferumoxytol-enhanced MRI were in line with histological findings. In fact, expression of cyclooxygenase-1, microsomal-prostaglandin-E2 synthase-1, and macrophages was significantly lower in the aspirin group than the control group. Therefore, the study provided evidence that aspirin may attenuate the inflammatory process in the wall of human cerebral aneurysms and that ferumoxytol-enhanced MRI may reliably measure the effects of anti-inflammatory pharmacological interventions on IAs.

## 5. Ferumoxytol-Enhanced MRI in AVMs

Extrapolating from studies on IAs, we investigated in collaboration with the University of California, San Francisco the feasibility of imaging macrophages within intracranial AVM nidus using ferumoxytol-enhanced MRI. As such, four patients were imaged at baseline and at either one day or five days after infusion of 5 mg/kg of ferumoxytol. AVMs imaged at five days showed some intravascular tracer but had signal loss in the nidal region consistent with ferumoxytol localization. However, imaging at one day did not provide any useful information because residual intravascular ferumoxytol obscured evaluation for uptake in AVM vascular walls and stroma. Histological analysis showed pronounced CD68 staining in the AVM walls. This proof-of-principle study suggested that ferumoxytol-enhanced MRI may be used for assessing AVM inflammatory cell burden in general and macrophage infiltration in particular. As for IAs, the technique could potentially provide important information regarding the risk of AVM rupture or be used to monitor the effects of antinflammatory drugs on these lesions.

This study has also highlighted important limitations of ferumoxytol-enhanced MRI in AVMs. Because of the high blood volume of the nidus with its dense vascular network, detecting subtle changes in MRI signal can be difficult. In fact, it may be impossible to differentiate ferumoxytol particles that remain in the intravascular compartment from those phagocytosed by macrophages in AVM walls. For this reason, sufficient time should be given to allow clearance of intravascular ferumoxytol, and thus imaging at 24 h after ferumoxytol infusion is not recommended. Instead, imaging at a later time point, *i.e.*, at five days provides more informative data. Also, it may be useful to infuse a small dose of ferumoxytol and scan patients immediately, to test how the intravascular compartment appears initially without vascular wall signal loss. Subtraction images (5-day scan minus immediate scan) could prove valuable for this purpose. Future studies will be needed to demonstrate colocalization of ferumoxytol within macrophages of AVM walls and to optimize dose and timing parameters. 

## 6. Future Perspectives

Ferumoxytol-enhanced MRI is a very promising method for assessing inflammation in the walls of IAs and AVMs and for studying the natural course of the disease. Our future work will focus on quantification of the MRI signal loss, which will allow more objective assessment on the imaging findings. Thresholds for signal loss correlating with the risk of IA or AVM rupture may also be developed. A large, prospective, multicenter study to confirm whether ferumoxytol-enhanced MRI predicts aneurysm rupture will be conducted. 

Another potential application of ferumoxytol-enhanced MRI is the identification of the source of hemorrhage in patients harboring multiple IAs. In fact, it may be hard to determine which aneurysm has ruptured in this setting especially when the findings of head CT are negative or inconclusive [73]. This would avoid leaving unsecured those aneurysms that ruptured or unnecessarily treating unruptured aneurysms. A recent interesting study by Matouk *et al.* [74] has shown that high-resolution magnetic resonance vessel wall imaging may identify the site of rupture in patients with aneurysmal SAH, including those patients harboring multiple intracranial aneurysms. Lastly, because inflammation plays a major in the pathophysiology of vasospasm, ferumoxytol-enhanced MRI may have potential applications in this setting [75].

## 7. Conclusions

By detecting the activity of macrophages in aneurysm walls, ferumoxytol-enhanced MRI may identify unstable IAs at risk of rupture and can be used to monitor the effects on antinflammatory agents. In AVMs as well, ferumoxytol-enhanced MRI can be used for assessment of the activity of the inflammatory response. This technique holds promise for millions of patients harboring unruptured IAs and AVMs. Future improvements in technique and MRI signal quantification will certainly pave the way for widespread and efficient use of ferumoxytol-enhanced MRI in clinical practice. Meanwhile, there is little doubt that ferumoxytol-enhanced MRI has emerged as the most promising and exciting imaging tool for guiding the management of cerebrovascular lesions.

Our results with ferumoxytol for targeting macrophages as markers for inflammation in the vessel wall of cerebrovascular lesions are promising and should constitute the basis for further research. Although several questions require more investigation (such as when and how to best image macrophages), we are confident that the technique will have important applications in the future.
